# Rapid and selective quantitative colourimetric analysis of nitrite in water using a S-Nitrosothiol based method

**DOI:** 10.1016/j.wroa.2024.100265

**Published:** 2024-10-11

**Authors:** E. Latvyte, A. Greenwood, A. Bogush, J.E. Graves

**Affiliations:** a Centre for Manufacturing and Materials, Coventry University, Beresford Avenue, Coventry, UK; b School of Life Sciences, Coventry University, Whitefriars Street, Coventry, UK; c Centre for Agroecology, Water and Resilience, Ryton Organic Gardens, Coventry, UK

**Keywords:** Colourimetric detection, Nitrite, Wastewater, Water analysis

## Abstract

This study introduces a novel S-nitrosothiol based method for the rapid and highly selective detection of nitrite in complex water matrices. Sodium 3-mercapto-1-propanesulfonate forms a distinctive pink S-nitrosothiol compound upon interaction with nitrite in acidic media, allowing both visual and quantitative detection. Various factors affecting the absorbance of the final product were investigated, including pH, reaction time, acid type, and sodium 3-mercapto-1-propanesulfonate concentration. UV–Vis spectrophotometric analysis demonstrated an excellent linear correlation (R^2^ = 0.99) across a broad detection range (0.05 to 80 mmol l^-1^), while showing no interference from common ions such as nitrate or dissolved organic matter, a limitation frequently observed in conventional UV-based nitrite detection methods. The assay was further adapted into a pellet form to simplify field use, operating effectively at room temperature with a low detection limit (1.4 ppm). The S-nitrosothiol based method represents a safer and more environmentally friendly option for nitrite detection and shows a promising potential as a valuable addition to both field and laboratory water testing kits for nitrite analysis.

## Highlights

• A novel colorimetric method for nitrite detection was developed.
• Nitrite detected by formation of a pink S-nitrosothiol compound.
• Broad detection range from 0.05 to 80 mmol l^-1^.
• Adaptation of the assay into a pellet form for convenient field application.
• Excellent selectivity with no interference from common ions, including nitrate.

## Graphical abstract

**Figure:**
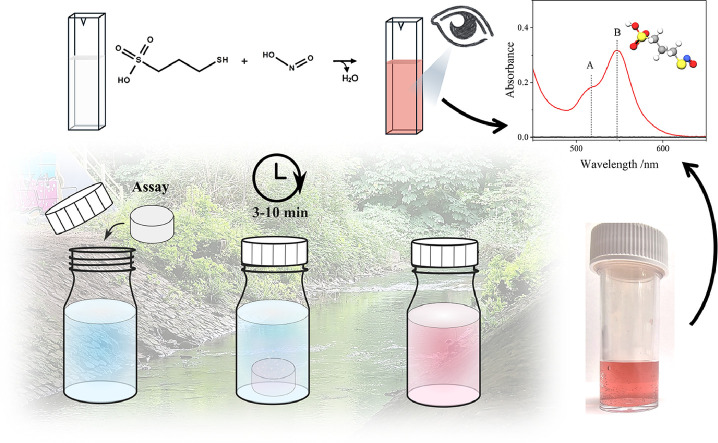

## 1 Introduction

Nitrite (NO_2_^-^) detection is important in wastewater, drinking water, and natural surface waters. In rivers, lakes, and other natural bodies, agricultural runoff is a primary source of nitrite contamination, which can severely disrupt aquatic ecosystems. The widespread use of nitrogen-containing fertilisers in agriculture, though beneficial for increasing crop yields (Sharpley, 2018), are a source of water-soluble nitrite and nitrate compounds that can lead to contamination of water and food supplies (Fan, 2014; Girkin and Cooper, 2022). In wastewater, nitrite levels are indicators of biological treatment efficacy. High nitrite concentrations can signal issues in the treatment system, such as incomplete nitrification or denitrification (Thakur and Medhi, 2019), and require corrective measures to ensure effective treatment.

Given the health and environmental concerns associated with nitrite contamination, there is a growing demand for accurate, selective, and cost-effective methods for detecting nitrites. Various methods have been introduced for nitrite detection, such as spectroscopic methods, ion chromatography (Ito et al., 2005), gas chromatography-mass spectroscopy (Tsikas et al., 2010) and high-performance liquid chromatography (Tatarczak-Michalewska et al., 2019). While the methods are exceptionally accurate, they require costly equipment which is often non-portable and can be time-consuming. UV detection of nitrite, typically in the 200–230 nm range, also suffers from low selectivity, as many organic compounds and nitrates absorb UV light, leading to interference and unreliable results (Bravo et al., 2009; Gentle et al., 2011).

The Griess assay, a well-established method in the water industry for detecting nitrite, involves a two-step reaction: NO_2_^−^ reacts with sulphanilamide to produce a diazonium salt intermediate, which then reacts with N-1-naphthylethelene diamine to produce an azo product that can be spectroscopically analysed at 540 nm (Filgueiras et al., 2021; Griess, 1879; Váradi et al., 2019). While widely used, the Griess method is prone to interference and has limited sensitivity, which has driven the development of modified versions of the assay (Lau and Wong, 2019). Griess assays have a very low and often insufficient upper detection limit of only 20 ppm, which can lead to inaccuracies due to over dilution when working with higher concentration samples (Hetrick and Schoenfisch, 2009; Sun et al., 2003). The modified assays often suffer from false positives from Fe^3+^, I^-^, S^2-^, and Cu^2+^ ions which can lead to low accuracy and limited use in complex matrices (Kafle, 2020; Wang et al., 2017). Lastly, Griess-based assays often require hazardous or unstable reagents (Hakobyan et al., 2022; Yardımcı, 2023).

In contrast to traditional UV methods, which exhibited significant interference from dissolved organic matter (DOM), nitrate (NO_3_^-^), and iron (Fe^3+^), leading to unreliable results, the proposed colourimetric method showed no such interference, ensuring more accurate nitrite detection, particularly in complex water samples. Importantly, it is demonstrated that this method can be adapted into a pellet form, enabling portability and ease of use in field applications without the need for sophisticated laboratory equipment. To demonstrate the practical application of the nitrite detection assay, it was assessed by analysing nitrite concentration in real samples from a municipal water source, local river, canal, eutrophic pond, and nitrified urine. This novel method outperforms existing techniques by combining high sensitivity, a broad detection range, simplicity of operation, and cost-effectiveness. It addresses the need for a safer, reliable and accessible nitrite detection method, suitable for widespread environmental monitoring.

## 2 Results and discussion

### 2.1 Sensing mechanism

**Fig. 1:**
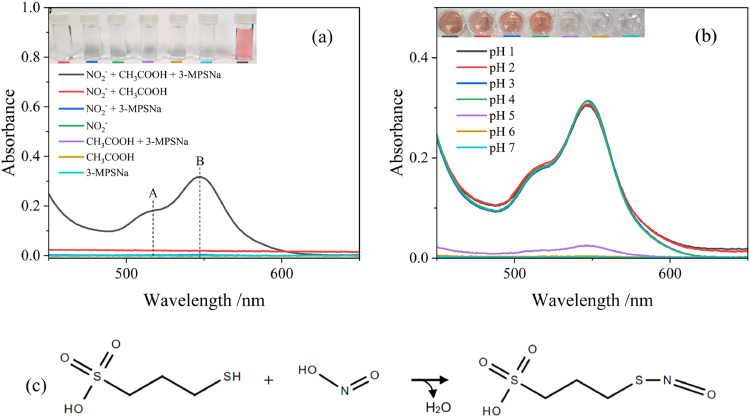
(a) Visible spectra of the 3-MPSNa nitrite colourimetric detection assay compared to various combinations of reagents. Inset shows a digital image of the corresponding solutions. (b) Effect of the pH on the visible spectrum for the assay. (c) Proposed assay reaction mechanism.

To determine the assay's sensing mechanism, the absorbance was measured for various combinations of the reagents used in the assay preparation by UV–Vis spectroscopy. This involved systematically varying the chemical components in order to observe how each combination affected the absorbance spectrum. As shown in Fig. 1(a), no peaks in the wavelength range of 450–650 nm were observed for any combination of 20 mmol l^-1^ sodium 3-mercapto-1-propanesulfonate (3-MPSNa), 1 mol l^-1^ CH_3_COOH, and 20 mmol l^-1^ NO_2_^-^ sample without the standard combination of all three materials. None of the described samples produced a visual colour change. With the correct combination of the three reagents, two peaks were observed at 517 nm (A) and 547 nm (B). The peak absorbance ratio was approximately A_517_/A_547_ = 0.57. In addition, the inset shows the digital image of the 3-MPSNa assay with and without addition of NO_2_^-^ which shows that a significant colour change to bright pink occurred when the three reagents were used.

### 2.2 Optimising the reagents

To optimise the performance of the assay, pH, acid type, concentration of 3-MPSNa, and reaction time were investigated. Initial experiments, shown in Fig. 1(a), demonstrated that the NO_2_^-^ + 3-MPSNa solution, in the absence of CH_3_COOH, did not yield any colour change or absorbance signal. The results indicated that the non-acidified assay is not suitable for nitrite detection and the pH of the buffer solution was an important factor in the reaction mechanism. To study the effect of pH on the assay performance, a series of the assay solutions with pH values, ranging between 1 and 7, were prepared. As shown in Fig. 1(b), at pH 6 and 7, no visible colour change was seen, and no peaks appeared when analysed by UV–Vis. At pH 5, a light pink colour developed, with two peaks at 517 nm and 547 nm (A_547_ = 0.025). With decreasing pH, the colour of the assay became more intense, and the two absorption peaks increased significantly (A_547_ = 0.30). The absorbance remained stable at A_547_ = 0.30 ± 0.02 across pH values of 1–4, confirming the assay is effective when the pH ≤ 4.

The specific reaction mechanism for the developed assay can be assigned to the two-step formation of *S*-nitrosothiol (R-S-*N*=*O*) (Möller and Denicola, 2022), Fig. 1(c). In the first step, nitrous acid (HNO_2_) is formed by reaction of NO_2_^-^ and the selected acid, Eq. (1):

(1) NO_2_^-^ + *H*^+^ = HNO_2_

In the second step, the 3-MPS reacts with HNO_2_ to form 3-(nitrososulfanyl)propane-1-sulfonic acid according to the following Eq. (2):

(2) HSCH_2_CH2CH_2_SO_3_H + HNO_2_ → NOSCH_2_CH2CH_2_SO_3_H + H_2_O

**Fig. 2:**
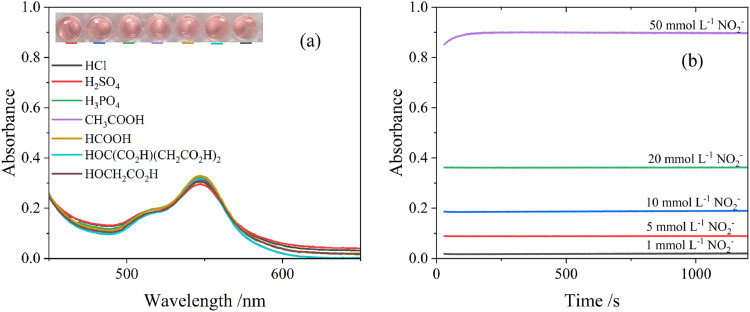
(a) Effect of acid type on visible spectrum for nitrite assay. Parameters for all experiments in (a-c) were *T* = 20 °C, [NO_2_^-^] = 20 mmol l^-1^, [3-MPSNa] = 20 mmol l^-1^, [acid] = 1 mol l^-1^. (b) Effect of the time on the absorbance at 547 nm in presence of [3-MPSNa] = 50 mmol l^-1^, [CH_3_COOH] = 1 mol l^-1^ and [NO_2._] = 1, 5, 10, 20, 50 mmol l^-1^, *T* = 20 °C.

To determine the effect of the acid used for the assay, HCl, H_2_SO_4_, H_3_PO_4_, CH_3_COOH, HCOOH, HOC(CO_2_H)(CH_2_CO_2_H)_2_ and HOCH_2_CO_2_H were tested for acidification. For the experiment, 1 mol l^-1^ of acid of interest was added to the solution, Fig. 2(a). It can be seen the typical bright pink colour was produced in all cases and the type of the acid did not have an impact on the reaction. The test has also shown that PO_4_^3-^, Cl^-^, SO_4_^2-^, CH_3_COO^-^, HCOO^-^, C_2_H_3_O_3_^-^ and C_6_H_7_O_7_^-^ ions did not interfere with the reaction, with consistent absorption values of 0.31 ± 0.02 at 547 nm. For standardisation of further tests, the addition of 1 mol l^-1^ CH_3_COOH was chosen for pH adjustment of the assays due to low cost, broad availability, as well as ease and safety of handling.

Optimal reaction time was also an important part of producing an accurate result. The assay of the 20 mmol l^-1^ 3-MPSNa + 100 mmol l^-1^ KNO_2_ acidified with 1 mol l^-1^ CH_3_COOH (pH = 1.85) was prepared and assessed. The absorbance at 547 nm was continuously analysed from 30 to 1200 s and the values were plotted in Fig. 2(b). Initially, a slight change in absorbance from 0.85 to 0.90 was observed in the first 250 s for the 50 mmol l^-1^ sample, while the other tested concentrations remained stable throughout the experiment. The colourimetric response reached equilibrium at 260 s and then remained stable for the duration of the 20 min experiment. The reaction time of 300 s (5 min) was chosen for further studies to ensure all samples have reached the maximum absorbance during analysis.

**Fig. 3:**
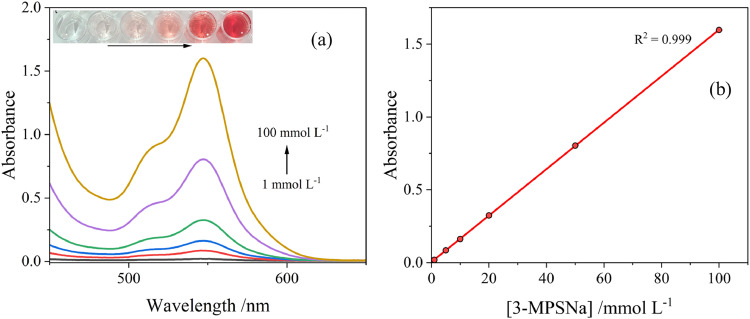
(a) Effect of 3-MPSNa concentration on UV–Vis absorption spectrum recorded in [NO_2_^-^] = 100 mmol l^-1^, [CH_3_COOH] = 1 mol l^-1^, [3-MPSNa] = 1, 5, 10, 20, 50, 100 mmol l^-1^, *T* = 20 °C. Inset shows a digital image of the assays. (b) Plot of peak absorbance values against 3-MPSNa, showing a linear relationship.

The effect of concentration of the 3-MPSNa for accurate detection of nitrite was also investigated. As shown in Fig. 3(a), with 1 mmol l^-1^ of 3-MPSNa added to the sample in the presence of acidified 100 mmol l^-1^ of KNO_2_, no detectable change in colour or spectra was observed. A light pink colour and a peak at 547 nm was observed when the concentration was increased to 5 mmol l^-1^. Increasing the concentration to 10 mmol l^-1^ resulted in a brighter pink colour, and at 20 mmol l^-1^, the transition from light pink to coral pink was clear. The most intensive colour change was observed when the concentration reached 50 and 100 mmol l^-1^, with a deepening in the colour and a two-fold increase in the absorption peak at 547 nm from 0.8 to 1.6. Overall, the absorption peak increased linearly (R^2^ = 0.999) with increasing 3-MPSNa concentration, Fig. 3(b). For consistency, the 3-MPSNa concentration was kept as 100 mmol l^-1^ for further studies.

### 2.3 Quantitative analysis

**Fig. 4:**
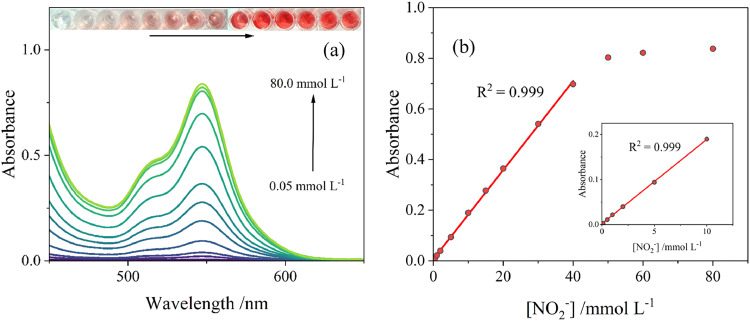
(a) Effect of nitrite concentration on UV–Vis absorption spectrum recorded in [3-MPSNa] = 100 mmol l^-1^, [CH_3_COOH] = 1 mol l^-1^ and [NO_2_^-^] = 0, 0.05, 0.1, 1, 2, 5, 10, 15, 20, 30, 40, 50, 60, 80 mmol l^-1^. *T* = 20 °C. (b) Plot of peak absorbance values against NO_2_^-^ concentrations.

To determine if the assay has practical use, its ability to quantitively detect nitrite in given samples is critical. In this work, the nitrite detection accuracy was determined by absorbance kinetic studies under the determined optimal working conditions. Visual colour and absorbance response were monitored in the NO_2_^-^ concentration range of 0.05–80 mmol l^-1^, shown in Fig. 4(a). As previously shown, no peak is observed at 547 nm when no NO_2_^-^ is added to the assay system. With an addition of 0.05 mmol l^-1^ of NO_2_^-^, a small peak formed at 547 nm which signified the presence of NO_2_^-^. The limit of detection (LOD) was determined to be 0.03 mmol l^-1^ (1.4 ppm) and the limit of quantification (LOQ) was 0.08 mmol l^-1^ (3.7 ppm), Table S1. The detection limit set is lower than the 3-ppm allowed limit set by World Health Organisation guidelines, suggesting the detection method has real practical application value for fresh and drinking water sources.

Upon analysis of the UV range (< 400 nm) (Figure S1), it was noted that the introduction of nitrite led to the formation of an absorbance peak at 332 nm (denoted as peak C). This peak is consistent with earlier findings, where nitrosation reactions involving thiols were shown to produce two peaks in the visible region as well as an UV absorbance peak at similar wavelengths due to the S–N bond's electronic transitions (Morakinyo et al., 2012). The peak at 332 nm exhibited a good linear relationship (R^2^ = 0.998) within the range of low concentrations (0.01 mmol l^-1^ – 1 mmol l^-1^), thus potentially facilitating the quantification of nitrite ions even in samples containing as little as 0.5 ppm.

With further increase in NO_2_^-^ concentration, the absorbance at 547 nm increased linearly for concentration ranges from 0.01 mmol l^-1^ to 10 mmol l^-1^ (R^2^ = 0.999) as well as from 0.05 to 40 mmol l^-1^ (R^2^ = 0.999), Fig. 4(b). This has suggested the assay has the potential ability to accurately determine NO_2_^-^ concentration for both, high and low concentrations. Above 40 mmol l^-1^, the absorbance values stopped increasing and no visible colour change was observed, indicating reaction saturation.

With increasing concentration of 3-MPSNa in the assay to 500 mmol l^-1^, the absorbance values for the NO_2_^-^ concentration of 50–80 mmol l^-1^ also increased and once again showed a linear relationship (R^2^ = 0.999), Figure S2. This meant the upper limit of quantification can be adjusted according to expected NO_2_^-^ values in the samples of interest. As conventional colourimetric nitrite detection methods operate at ranges between 0 and 3.5 mmol l^-1^ (Britschgi et al., 2020), the developed assay would likely be more suitable for higher-nitrite level water sources, such as wastewater, and for food manufacturing.

### 2.4 Selectivity and interference

Various ions can interfere with chemical reactions, leading to false positives in nitrite detection. The acid selection test has confirmed the 3-MPSNa-based nitrite test had a good selectivity towards NO_2_^-^ with no interference from PO_4_^-^, Cl^-^, SO_4_^-^, SO_3_^-^, NO_3_^-^, CH_3_COOH, HCOOH, HOC(CO_2_H)(CH_2_CO_2_H)_2_ and HOCH_2_CO_2_H.

**Fig. 5:**
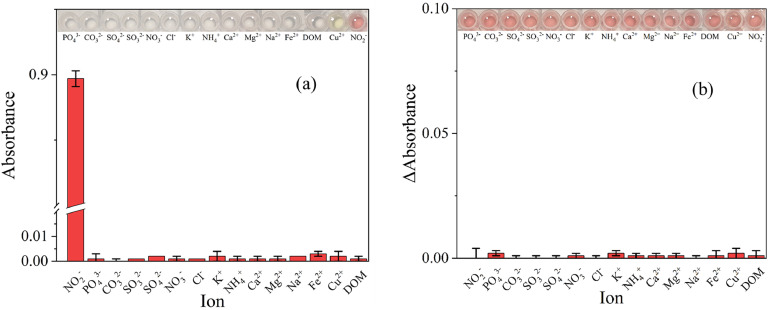
(a) Selectivity of the 3-MPSNa assay in presence of DOM and various commonly found ions. (b) The difference in absorbance of the assay with an addition of 50 mmol l^-1^ NO_2_^-^ and 50 mmol l^-1^ of common ions. *n* = 3.

To further assess the selectivity, it was studied with an addition of DOM and common ions. The ions tested were NO_3_^-^, PO_4_^3-^, CO_3_^2-^, Cl^-^, SO_4_^2-^, SO_3_^2-^, *K*^+^, Na^+^, Mg^2+^, Cu^2+^, Fe^2+^^,^ Ca^2+^. Digital images in Figs. 5(a) show no visible colour change even at higher concentrations (50 mmol l^-1^). It should be noted the solutions with copper had a slight blue-green tint; this is typically associated with Cu(II) cation-containing solutions (Leygraf et al., 2019), and it was not a result of a reaction with 3-MPSNa. Notably, the NO_3_^-^ ions did not react with acidified 3-MPSNa, allowing clear differentiation from NO_2_^-^.

Similarly, upon addition of the common ions to the 3-MPSNa assay, no significant change was observed at 547 nm (Fig. 5(b)). Overall, the selectivity and interference study confirmed the assay's exceptional selectivity towards NO_2_^-^.

To assess the effectiveness of nitrite detection methods, a comparative analysis was conducted between the S-nitrosothiol based colourimetric method and UV spectrophotometry. UV analysis typically measures absorbance between 200 and 230 nm and 300–360 nm (Bravo et al., 2009; Gentle et al., 2011; Wang et al., 2021). Although nitrite has a distinct peak near 200 nm, this range is complicated by other ions, including nitrate and DOM, which also absorb at similar wavelengths (Mašić et al., 2015; Spangenberg et al., 2021). Additionally, common constituents in water sources, such as iron (Fe^3+^), and copper (Cu^2+^), cause overlapping absorbance signals across the UV range (Turner and Miles, 1957; Zhang et al., 2014).

In this study, overlapping spectra in the 200–220 nm range made it difficult to accurately measure nitrite due to nearby peaks for NO_3_^-^ (200 nm) and NO_2_^-^ (210 nm), Figure S3(a). Furthermore, the spectrum overlapped with signals from Cu^2+^, Fe^3+^, SO_3_^2-^, and DOM, Figure S3(b). At higher concentrations, NO_3_^-^ and NO_2_^-^ exhibited additional peaks at 300 nm and 350 nm, Figure S3(c). However, interference from Fe^3+^ and DOM in this range further complicated the analysis, Figure S3(d). Despite advanced spectral deconvolution software, distinguishing these peaks remains challenging in real-world conditions, making UV analysis of nitrite unsuitable for complex water matrices. Conversely, the S-nitrosothiol based assay demonstrated no interference from these ions, offering a more precise and reliable method for nitrite detection.

The developed S-nitrosothiol detection method offers several advantages over traditional azo dye-based methods. Azo dye methods often have limited upper detection limit (up to 20 ppm), affecting nitrite detection accuracy. Moreover, released azo dyes can accumulate in aquatic environments, leading to carcinogenic and mutagenic effects on aquatic life and potential human health impact (Chequer et al., 2011; Hashemi and Kaykhaii, 2022; Liu et al., 2022). Additionally, the disposal of azo dye-containing waste adds significant costs for proper waste management to avoid environmental contamination.

In contrast, S-nitrosothiols are naturally occurring compounds that function as nitric oxide donors in biological systems (Möller and Denicola, 2022). They play essential roles in various physiological processes (Maron et al., 2013). Replacing azo dye-based nitrite detection systems with S-nitrosothiol based alternatives could reduce the environmental impact of water analysis without the need for costly waste disposal.

### 2.5 Nitrite detection in real samples

For the analysis of real samples, the assay was adapted into a pellet form to eliminate the need for dilution, simplifying the procedure and enhancing ease of use. To evaluate its practical application, both the pellet and liquid assays were used to analyse various water samples (Table 1). Samples were collected from: Coventry's municipal tap water, canal, eutrophic pond, the Sowe River valley, and nitrified urine, Figure S4. Total organic carbon (TOC) levels varied, with tap water containing <1 ppm, while the canal, river, pond, and urine samples had TOC concentrations of 19.3 ppm, 23.7 ppm, 36.9 ppm, and 58.3 ppm, respectively.

**Table 1:** Results for nitrite determination tests in real water samples.

Analyte	Added NO_2_^-^ /mmol l^-1^	Found NO_2_^-^*/mmol l^-1^		Recovery /%	RSD /% (*n* = 3)	
		Pellet	Liquid		Pellet	Liquid
Tap	0	< 0.03	<0.03	–	–	–
	1	0.98 ± 0.03	0.99 ± 0.01	98.4	2.66	0.93
	5	5.04 ± 0.08	5.01 ± 0.03	100.7	1.61	0.70
Canal	0	< 0.03	<0.03	–	–	–
	1	0.97 ± 0.03	0.97 ± 0.01	97.0	2.71	0.39
	5	5.12 ± 0.11	5.12 ± 0.02	102.5	2.16	0.39
River	0	< 0.03	<0.03	–	–	–
	1	1.02 ± 0.03	1.00 ± 0.01	101.5	2.59	0.81
	5	5.21 ± 0.14	5.21 ± 0.02	104.3	2.70	0.41
Pond	0	0.10 ± 0.01	0.10 ± 0.01	–	2.59	1.42
	1	1.09 ± 0.02	1.10 ± 0.01	97.6	1.84	0.60
	5	5.11± 0.04	5.10 ± 0.04	100.0	0.77	0.84
Nitrified urine	0	0.60 ± 0.01	0.60 ± 0.01	–	1.78	0.27
	1	1.59 ± 0.04	1.59 ± 0.01	98.9	2.52	0.41
	5	5.60 ± 0.05	5.61 ± 0.05	99.3	0.85	0.92

⁎ Average (*n* = 3).

Nitrite was undetectable in both tap water and canal samples, Figure S5(a),(b). A small absorption peak was observed in the Sowe River sample, though it was below confidence limits, Figure S5(c). The eutrophic pond sample showed a low nitrite level of 0.1 mmol l^-1^ (4.6 ppm), likely due to nitrification of ammonia in the nutrient-rich environment. Lastly, the nitrified urine samples showed the highest level of nitrite – 0.6 mmol l^-1^ (27.6 ppm). While the results of the developed S-nitrosothiol-based assay correspond well with those obtained using a conventional nitrite cuvette test (Table S2), it's worth noting that the cuvette test required sample dilution due to its limited detection range. In contrast, the developed assay did not require dilution, further enhancing its practicality in field applications, especially in high-concentration samples.

When analysed by the UV nitrite detection method, a nitrite peak in the pond sample was observed, consistent with a 4.6 ppm concentration, Figure S5(d),(e). However, for other samples, significant interference from ions and DOM was observed which obscured the nitrite peak and made accurate detection impossible. In contrast, the 3-MPSNa-based colourimetric assay exhibited minimal interference, thereby enabling reliable quantification of nitrite across diverse and complex water matrices. This highlights the assay's superior selectivity compared to UV spectrophotometry.

Furthermore, the samples that were spiked with 1 mmol l^-1^ and 5 mmol l^-1^ of nitrite demonstrated a clear correlation with the determined standard curve, with ≤ 2.71 % relative standard deviation (RSD) for the pellet assay, and 1.42 % for the liquid assay. These findings indicate that both the liquid and the pellet forms of the assay are effective for nitrite detection across a range of real water samples, demonstrating consistency in performance.

## 3 Conclusions

This study introduces a novel colourimetric method for nitrite detection in water, specifically optimised for field research. A one-step acidified 3-MPSNa assay was developed, achieving optimal performance at pH 1–4 with 3-MPSNa concentrations above 100 mM, offering a detection range from 1.4 to 3700 ppm for nitrite. This flexibility allows for precise adjustments in detection limits by varying reagent concentrations, enhancing its applicability to diverse environmental contexts. In comparison, UV-based detection methods demonstrated significant interference from dissolved organic matter, iron (III), nitrate, and sulphite ions, reducing their reliability in complex matrices. The colourimetric assay, in contrast, exhibited superior selectivity with minimal interference from these common contaminants.

Validation using real water samples, including river, canal, eutrophic pond, and nitrified urine, confirmed the method's high accuracy, closely aligning with conventional nitrite tests. The assay's versatility, minimal interference, and broad detection range make it an effective alternative to traditional methods, offering enhanced suitability for environmental monitoring. Additionally, the developed assay uses non- toxic chemicals, further reducing environmental impact and lowering waste disposal costs, making it both a safer and more sustainable option for large-scale applications.

## 4 Materials

### 4.1 Chemicals and reagents

All reagents used in this study were analytical grade, unless specified otherwise. KNO_2_, NaNO_3_, FeSO_4_∙7H_2_O, Fe(NO_3_)_3_, FeCl_3_ (NH_4_)_2_SO_4_, CuCl_2_, MgSO_4_, Na_2_CO_3_, Na_2_SO_3_, and HCl were purchased from Fisher Scientific, UK; H_2_SO_4_, HNO_3_, CH_3_COOH (acetic acid), HCOOH (formic acid), HOC(CO_2_H)(CH_2_CO_2_H)_2_ (citric acid), Na_2_SO_3_, HOCH_2_CO_2_H (glycolic acid), H_3_PO_4_, and sodium 3-mercapto-1-propanesulfonate (3-MPSNa, technical grade), were purchased from Sigma Aldrich, UK. All solutions were prepared with ultrapure water (18.2 MΩ∙cm, Elga Purelab Chorus 1, UK).

### 4.2 Instruments

UV–Vis absorption spectra were analysed by a double-beam UV–Vis spectrophotometer (UV-2450, Shimadzu, UK) between 300 nm and 650 nm. The pH of the solutions was adjusted with CH_3_COOH and measured by a calibrated pH meter (Orion Star A121, Thermo Scientific, UK).

### 4.3 Assay optimisation

Prior to experiments, a 1 mol l^-1^ stock solution of 3-MPSNa was prepared and refrigerated at 4 °C before use. Nitrite stock solutions (1 mol l^-1^, 0.1 mol l^-1^ and 0.05 mol l^-1^) were freshly prepared on the day of the experiments by dissolving the required amount of KNO_2_ in ultrapure water. The NO_2_^-^ standards were then prepared by performing serial dilutions to achieve the required concentrations (0.01, 0.02, 0.05, 0.1, 0.2, 0.5, 1, 2, 5 10, 20, 50, 60, 80 mmol l^-1^).

The sensing assay was prepared using the following reagents – 20 mmol l^-1^ KNO_2_, 1 mol l^-1^ CH_3_COOH and 20 mmol l^-1^ 3-MPSNa. The assay was separately optimised by determining the effect of acid used to adjust the pH of the solutions by an addition of 1 mol l^-1^ of one of the following: HCl, H_2_SO_4_, H_3_PO_4_, CH_3_COOH, HCOOH, HOC(CO_2_H)(CH_2_CO_2_H)_2_, or HOCH_2_CO_2_H. Optimal detection time was determined by preparing an assay and monitoring the absorbance at 547 nm in kinetic mode between 30 - 1200 s by UV–Vis spectrophotometer.

The effect of the pH was studied by adjusting the pH of the solution to pH 1–7 with CH_3_COOH (or HCl for the pH 1 solution) in the presence of 20 mmol l^-1^ KNO_2_ and 20 mmol l^-1^ 3-MPSNa, while keeping the other factors constant. The optimal 3-MPSNa concentration was determined by studying the effect of the concentration (1 - 500 mmol l^-1^) in the presence of 100 mmol l^-1^ KNO_2_ and 1 mol l^-1^ CH_3_COOH, while keeping the other factors constant.

The selectivity of the assay and the interference of various ions were studied by performing UV–Vis analysis of the 100 mmol l^-1^ 3-MPSA + 1 mol l^-1^ CH_3_COOH solutions (pH 1.85) in the presence of 50 mmol l^-1^ of PO_4_^3-^, CO_3_^2-^, SO_4_^2-^, SO_3_^2-^, Cl^-^, NO_3_^-^, *K*^+^, Na^+^, Mg^2+^, Cu^2+^, Fe^2+^and Ca^2+^ ions with and dissolved organic material with and without an addition of 50 mmol l^-1^ KNO_2_. The dissolved organic material stock sample was prepared by digesting humic acids, fulvic acids and tannins obtained from peat (Growmoor, UK). The solution was filtered through a 0.2 µm syringe filter (25 mm diameter nylon, VWR, UK) prior to usage.

The UV detection of nitrite was performed by analysing ultrapure water samples in presence of 1, 2, 20 and 50 ppm of NO_2_^-^. The interference of the UV method was studied by analysing ultrapure water samples in presence of 1, 2, 20 and 50 ppm of NO_3_^-^ as well as 10 ppm of Fe^3+^, Cu^2+^, SO_3_^2-^ and dissolved organic material. All experiments were performed at room temperature (20 °C).

### 4.4 Assay procedure

For sample analysis, two types of assays were prepared: liquid and solid pellet. The liquid assay stock was 1 mol l^-1^ 3-MPSA + 10 mol l^-1^ CH_3_COOH. To prepare a solid assay pellet, the liquid CH_3_COOH was substituted with crystalline HOCH_2_CO_2_H_._ The acid was selected due to its hygroscopic crystalline state to aid the formation of the solid and stable pellet. In detail, 0.14 g of 3-MPSNa and 0.38 g HOCH_2_CO_2_H were measured, transferred to a mortar and ground until a fine, homogenous powder was obtained. The resulting powder was then pressed into a pellet (Ø13 mm), Figure S6, by a manual hydraulic press set at 8 ton (15-ton press, evacuable pellet die, Specac, UK). This method enabled the assay to achieve its lowest limits of detection and eliminated the necessity for sample dilution.

To develop a standard curve for sample analysis, Figure S7, 0.1 mL of the assay stock solution or an assay pellet was added to 5 mL of the standard sample. The mixture was allowed to react for 5 min before the absorbance was measured at 547 nm. Each measurement was performed in triplicate to ensure accuracy. To determine limit of detection and limit of quantification, a series of 10 blank assay solutions were analysed at 547 nm and the values recorded. The limit of detection was calculated using the following Eq. (3):

(3) $$\text{LOD}=3.3\sigma_{0}$$

Where 3.3 is a numerical factor chosen according to the confidence level, σ_0_ is the standard deviation (S.D.) of a blank sample concentration measurement (*n* = 10) (Currie, 1999; Lister, 2005). Limit of quantification was calculated using the following Eq. (4):

(4) $$\text{LOQ}=10\sigma_{0}$$

Where 10 is a numerical factor chosen according to the confidence level (Currie, 1999; Lister, 2005).

### 4.5 Nitrite detection in real samples

Natural water samples were collected from the Coventry municipal water supply (tap), Sowe River, Coventry Canal, and eutrophic pond (Coventry). To prepare the nitrified urine sample, urine was collected from a single household, held for 14 days to undergo hydrolysis and nitrified in a 3 L reactor filled with porous ceramic rings (All Pond Solutions, UK). The nitrifying culture was introduced using a commercial nitrification product (API, UK). The sample selected for testing was sampled on day 5. The samples were collected in glass pre-cleaned bottles (acid-washed) and kept at 4 °C until analysis. Prior to analysis, the samples were first centrifugated at 4000 rpm and filtered through a 0.2 μm syringe filter (25 mm diameter nylon, VWR, UK). To determine the nitrite concentration, either 0.1 mL or the assay pellet was added to 5 mL of the water sample and allowed to dissolve and react for 5 min before recording the absorbance at 547 nm in triplicate. Finally, the samples were subsequently spiked with 1 and 5 mmol l^-1^ of NO_2_^-^ and the standard analysis procedure repeated. The measured values were used to calculate recovery (ratio of expected concentration to measured concentration) and relative standard deviation (RSD). Additional analysis of NO_2_^-^, NO_3_^-^, and TOC were performed with LCK342, LCK339, and LCK385, respectively (Hach Lange, UK). The cuvettes were analysed by a calibrated spectrophotometer (DR2800 Hach, UK). The samples were diluted to below the upper detection ranges of 6 ppm, 13.5 ppm, and 30 ppm concentration for the NO_2_^-^, NO_3_^-^ and the TOC test respectively.

## CRediT authorship contribution statement

**E. Latvyte:** Writing – review & editing, Writing – original draft, Validation, Resources, Methodology, Investigation, Formal analysis, Data curation, Conceptualization. **A. Greenwood:** Writing – review & editing, Methodology. **A. Bogush:** Writing – review & editing, Methodology. **J.E. Graves:** Writing – review & editing, Supervision, Resources, Funding acquisition.

## Declaration of competing interest

The authors declare the following financial interests/personal relationships which may be considered as potential competing interests:

Egle Latvyte, John Graves reports financial support was provided by 10.13039/501100000780European Commission. If there are other authors, they declare that they have no known competing financial interests or personal relationships that could have appeared to influence the work reported in this paper.

## Data Availability

Data will be made available on request.

## Appendix Supplementary materials

Image, application 1
