# Supplementary material for: Rapid and selective quantitative colourimetric analysis of nitrite in water using a S-Nitrosothiol based method
Source: Water Res X. 2024 Oct 11;25:100265. doi: 10.1016/j.wroa.2024.100265 (PMC11533637; doi:10.1016/j.wroa.2024.100265)
Supplement: Supplementary file 1 [file mmc1.docx]

**Supplementary Information**

Rapid and Selective Quantitative Colourimetric Analysis of Nitrite in Water Using a S-Nitrosothiol Based Method

^1^E. Latvyte, ^2^A. Greenwood, ^3^A. Bogush, ^1^J. E. Graves

*^1^Centre for Manufacturing and Materials, Coventry University, Beresford Avenue, Coventry, UK*

*^2^Faculty of Health & Life Sciences, Coventry University, Coventry, UK*

*^3^Centre for Agroecology, Water and Resilience, Ryton Organic Gardens, Coventry, UK*

## Figures


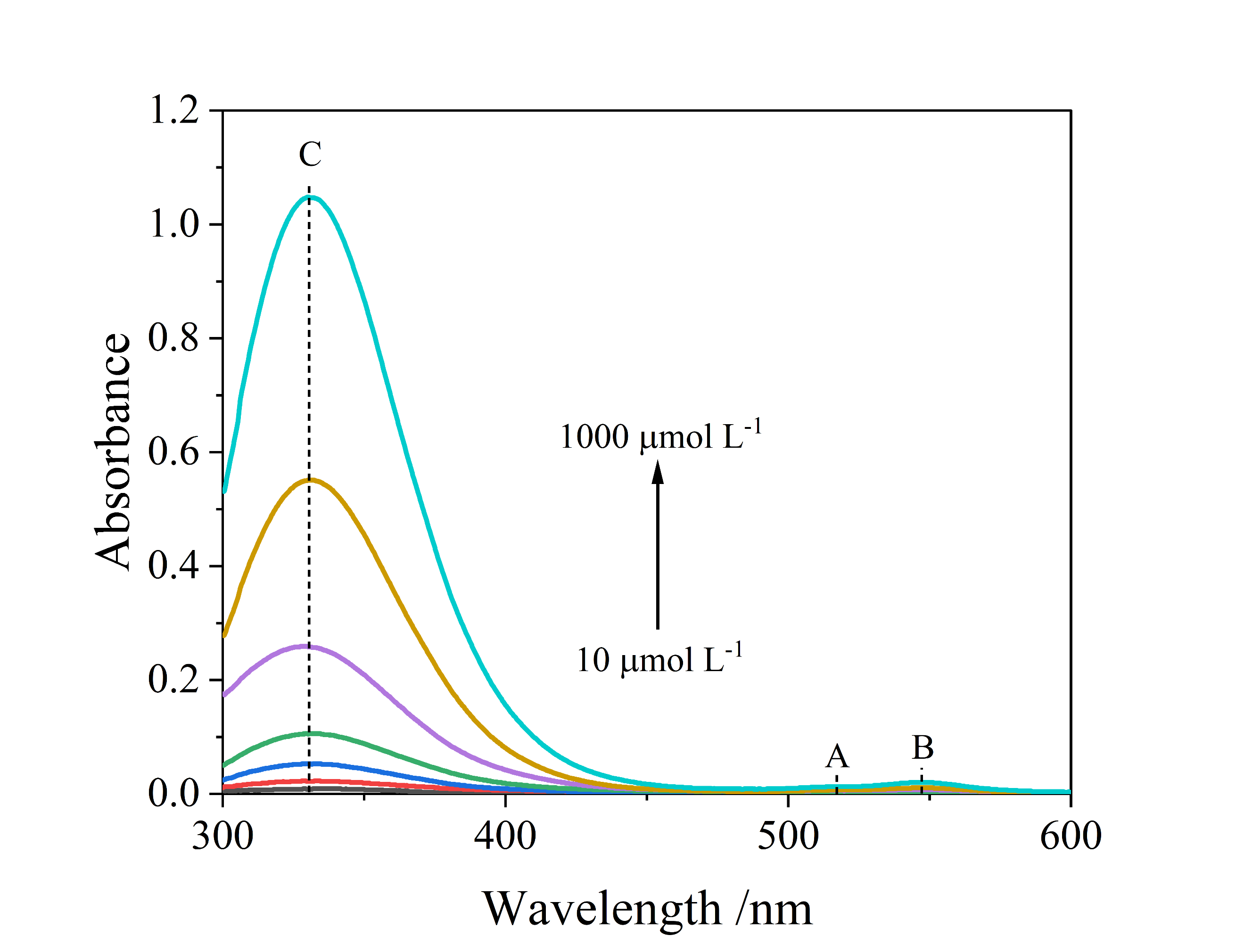

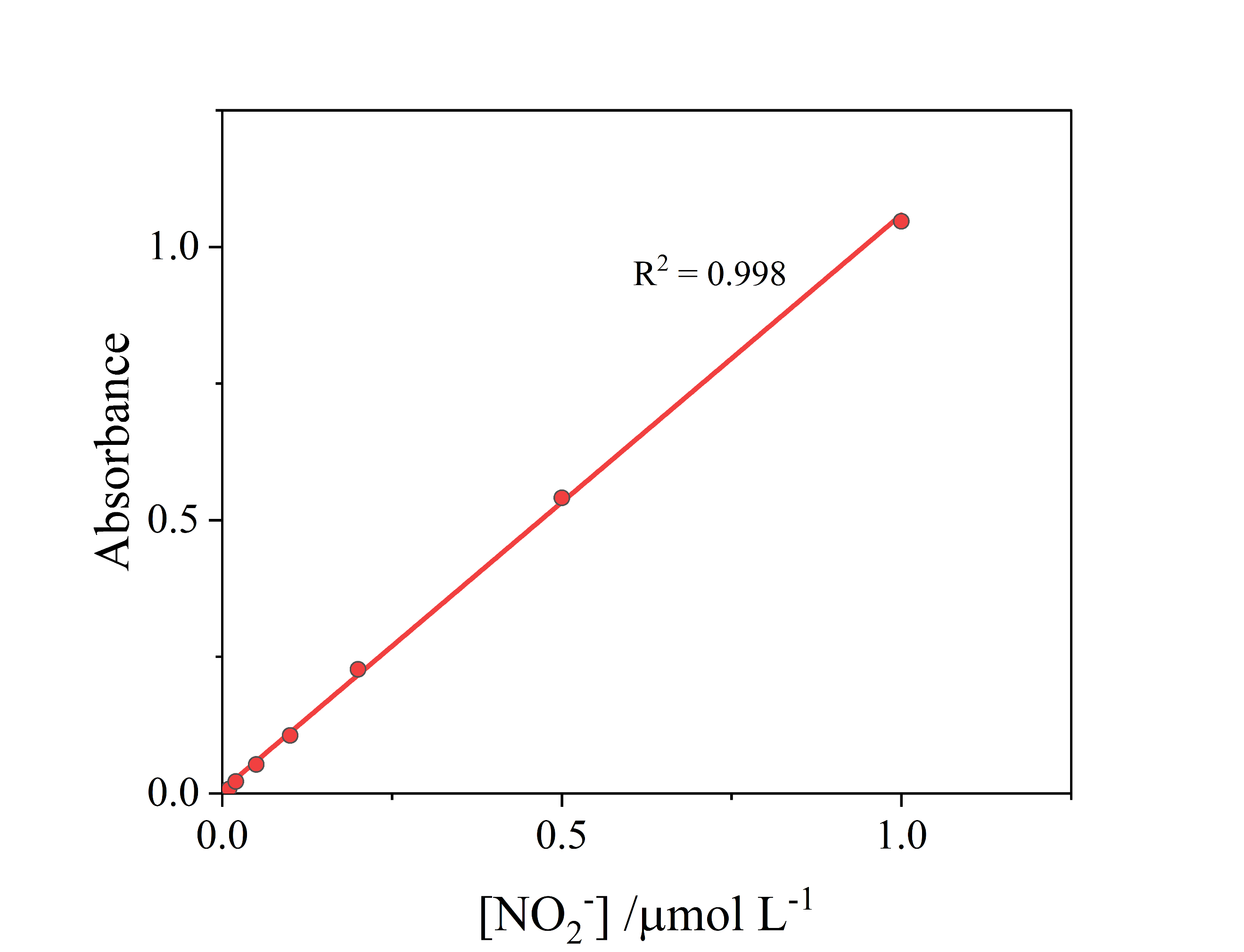


(a)

(b)

Figure S1. (a) Effect of NO_2_^-^ concentration on UV-Vis absorption spectrum recorded in [NO_2_^-^] = 10, 20, 50, 100, 200, 500, 1000 µmol L⁻¹, [CH_3_COOH] = 1 mol L^-1^, [3-MPSNa] = 100 mmol L⁻¹, T = 20°C. (b) Plot of peak absorbance values (at 332 nm) against [NO_2_^-^].


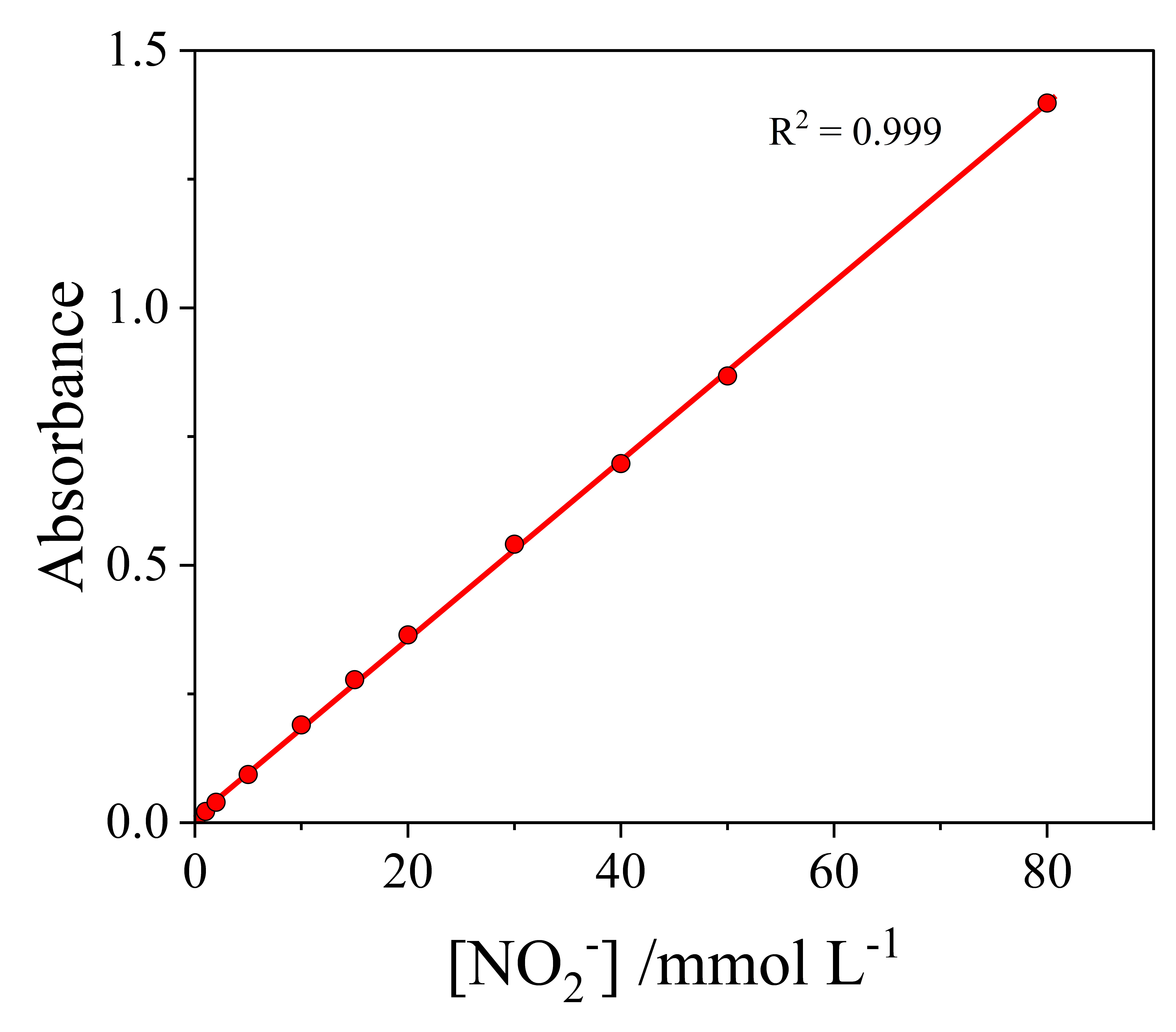


Figure S2. Plot of peak absorbance values against NO_2_^-^ concentrations, showing a linear relationship. Values obtained in 500 mmol L^-1^ 3-MPSNa + 1 mol L^-1^ CH_3_COOH assay.


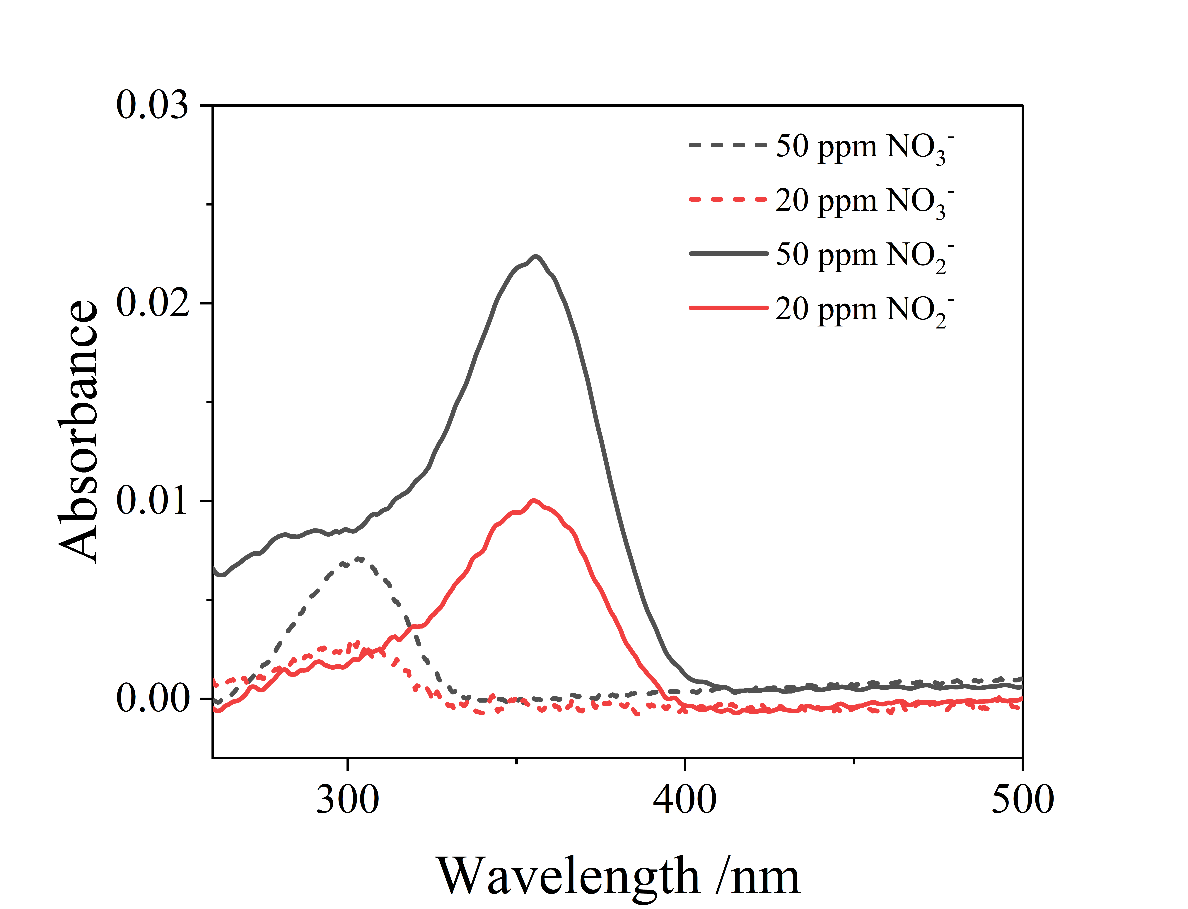

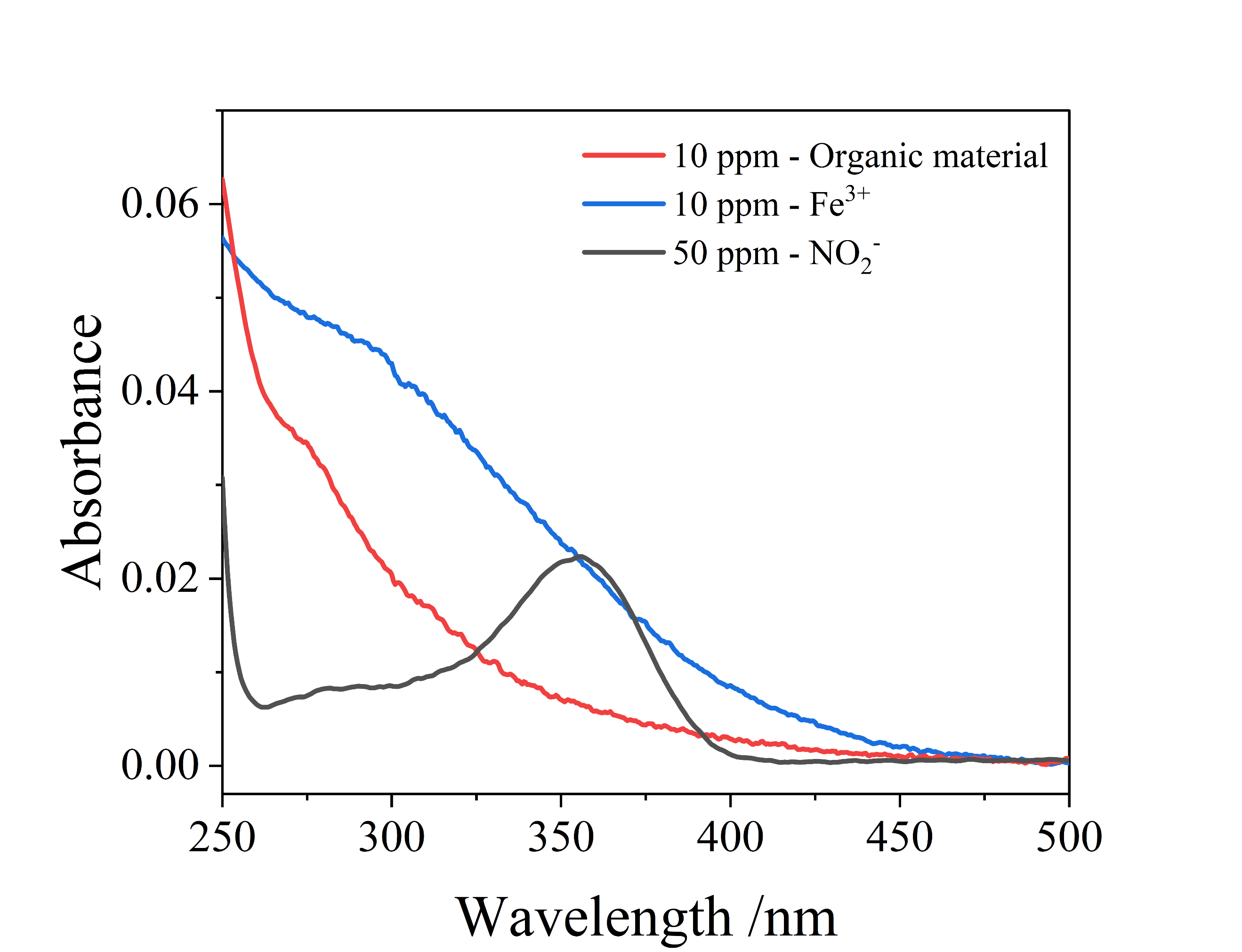

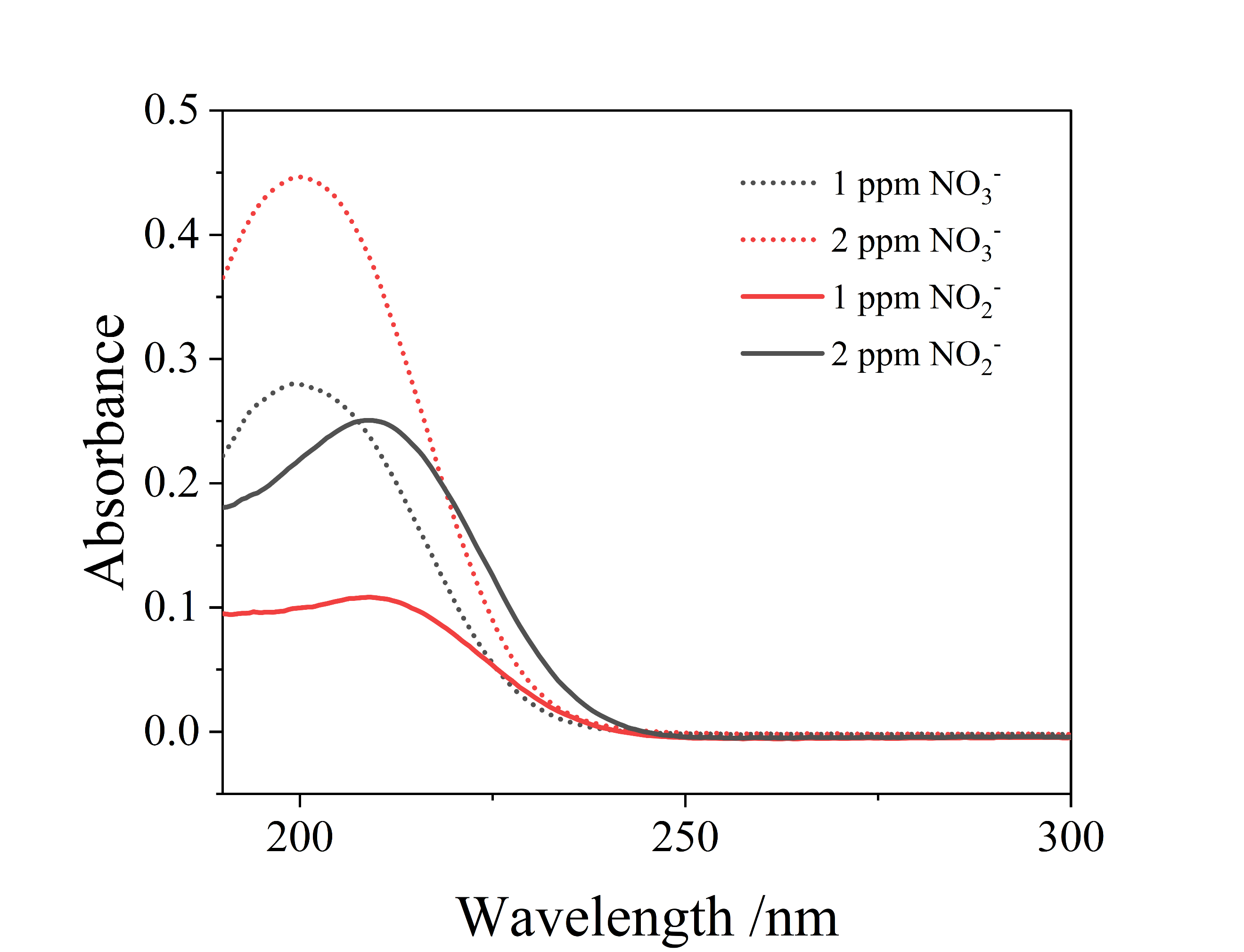

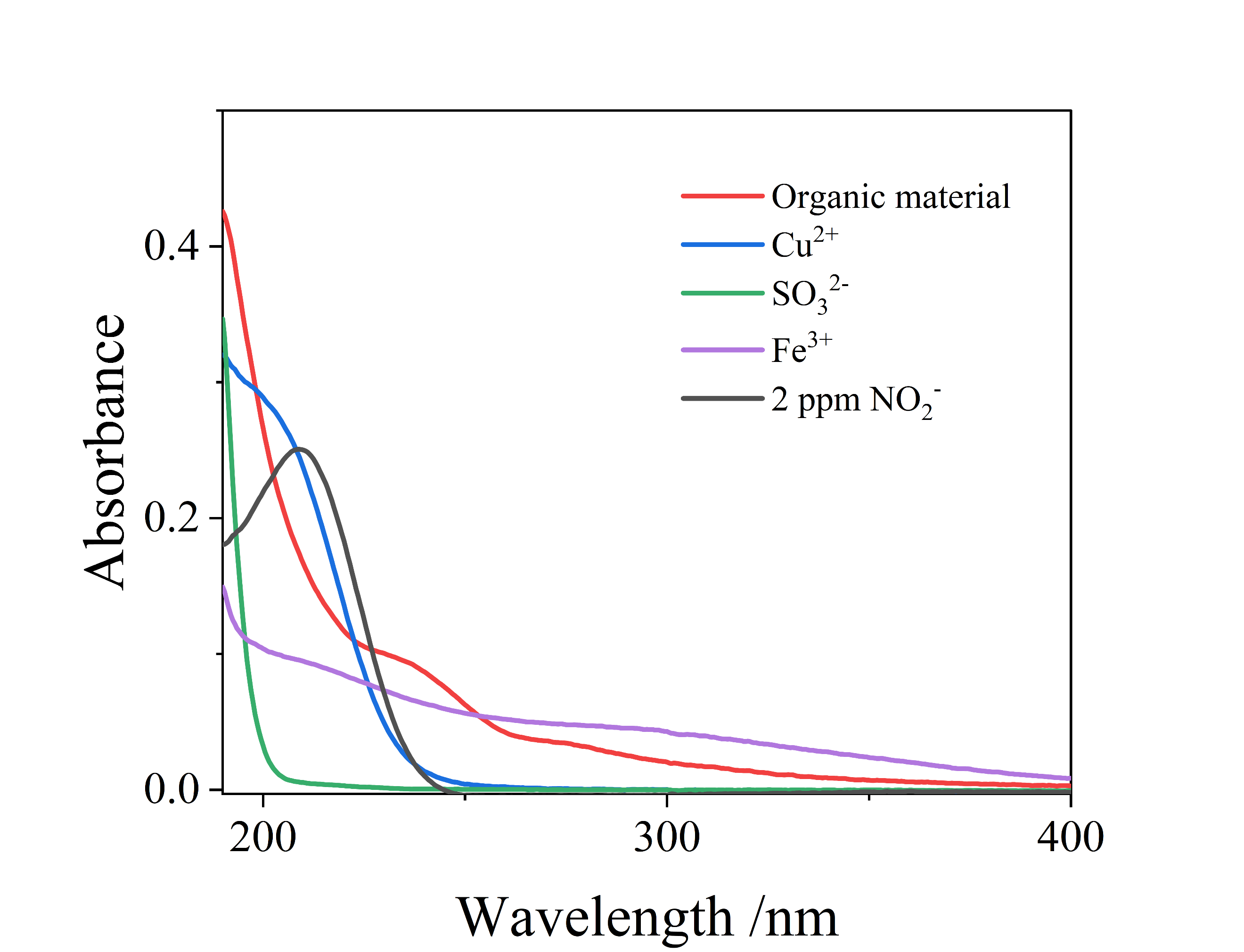


(a)

(b)

(c)

(d)

Figure S3. UV spectra of (a) NO_2_^-^ and NO_3_^-^ and (b) NO_2_^-^ and interfering ions (10 ppm) in water. UV-Vis spectra of (c) NO_2_^-^ and NO_3_^-^ and (d) NO_2_^-^ and interfering ions (10 ppm) in water.


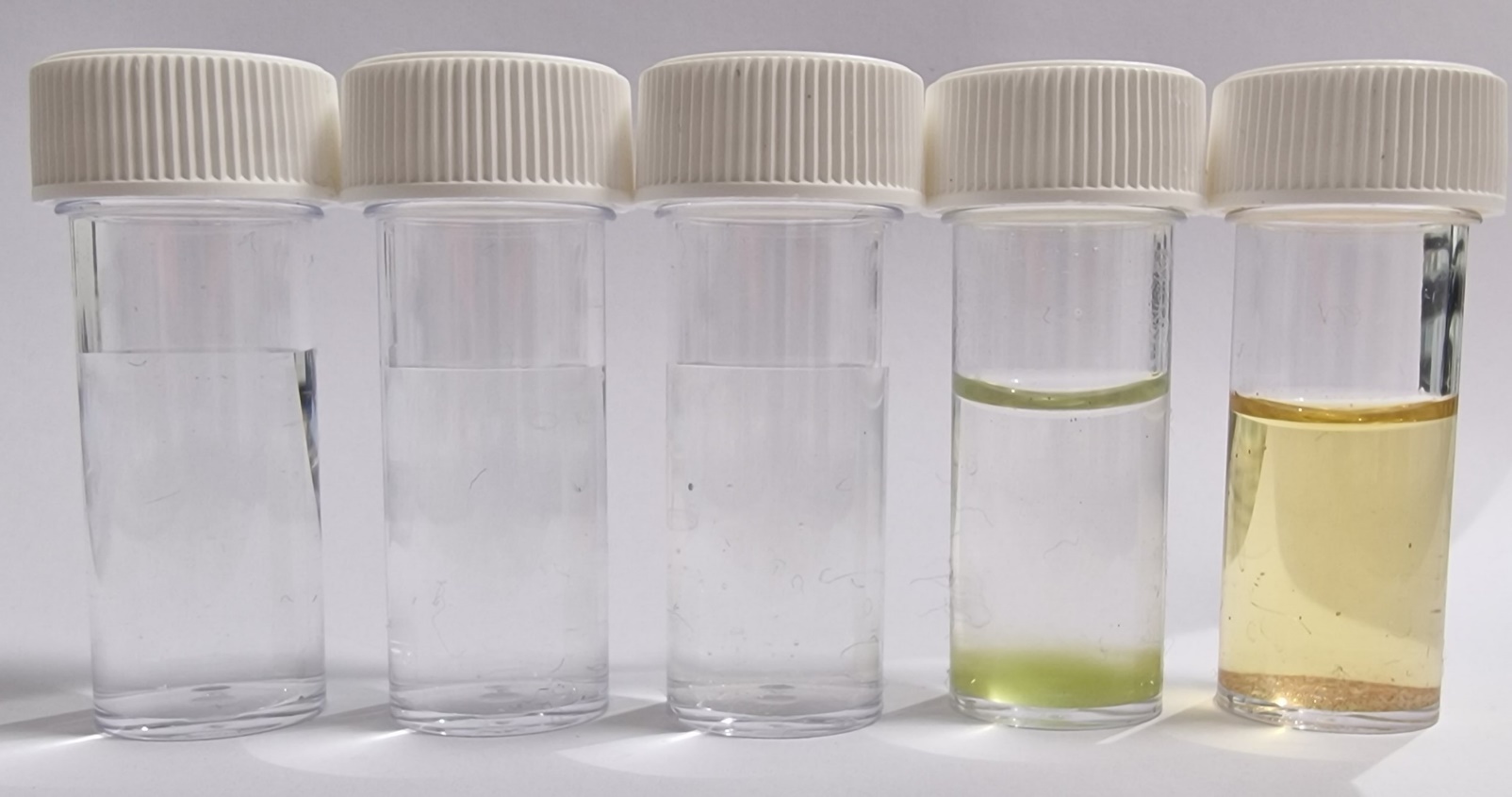


Figure S4. Real samples analysed in this work. From left to right – tap water, River Sowe, Coventry Canal, local eutrophic pond, nitrified urine samples.


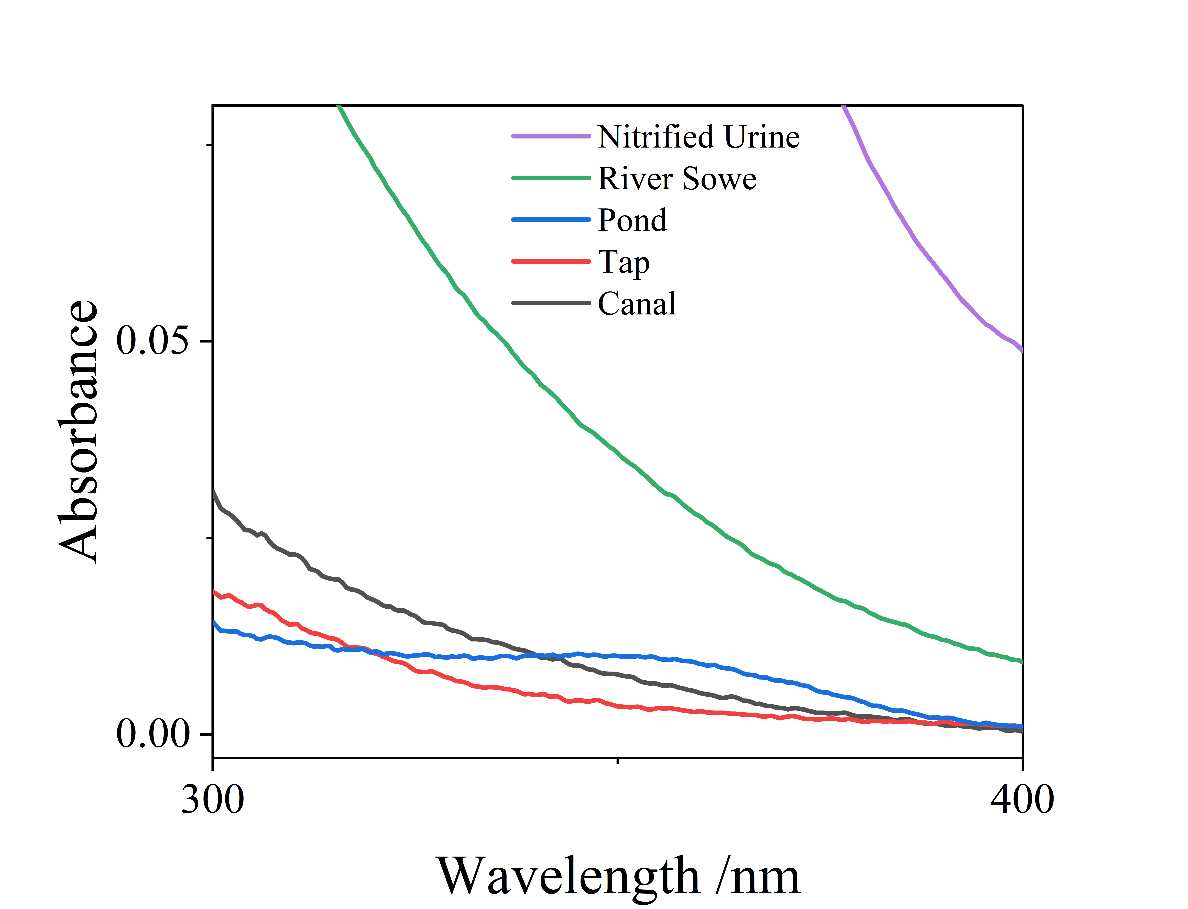

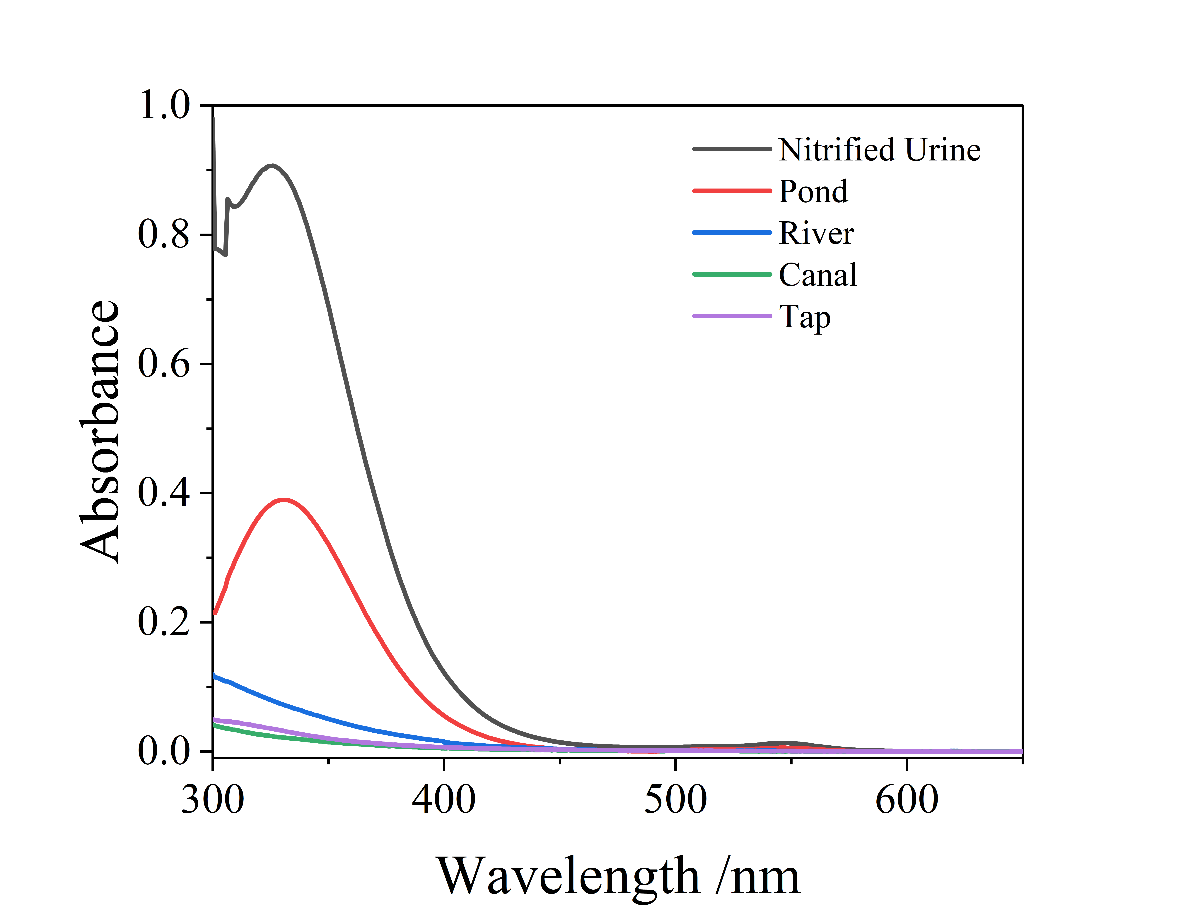


Figure S5. (a) UV-Vis and (b) magnified visible spectra of real unspiked samples obtained with 100 mmol L^-1^ 3-MPSNa + 1 mol L^-1^ HOCH_2_CO_2_H (glycolic acid) assay pellet. (c) Magnified visible spectrum of the river Sowe sample. (d) UV spectra of real samples without an addition of the assay. (e) Magnified UV detection region of real samples without assay.


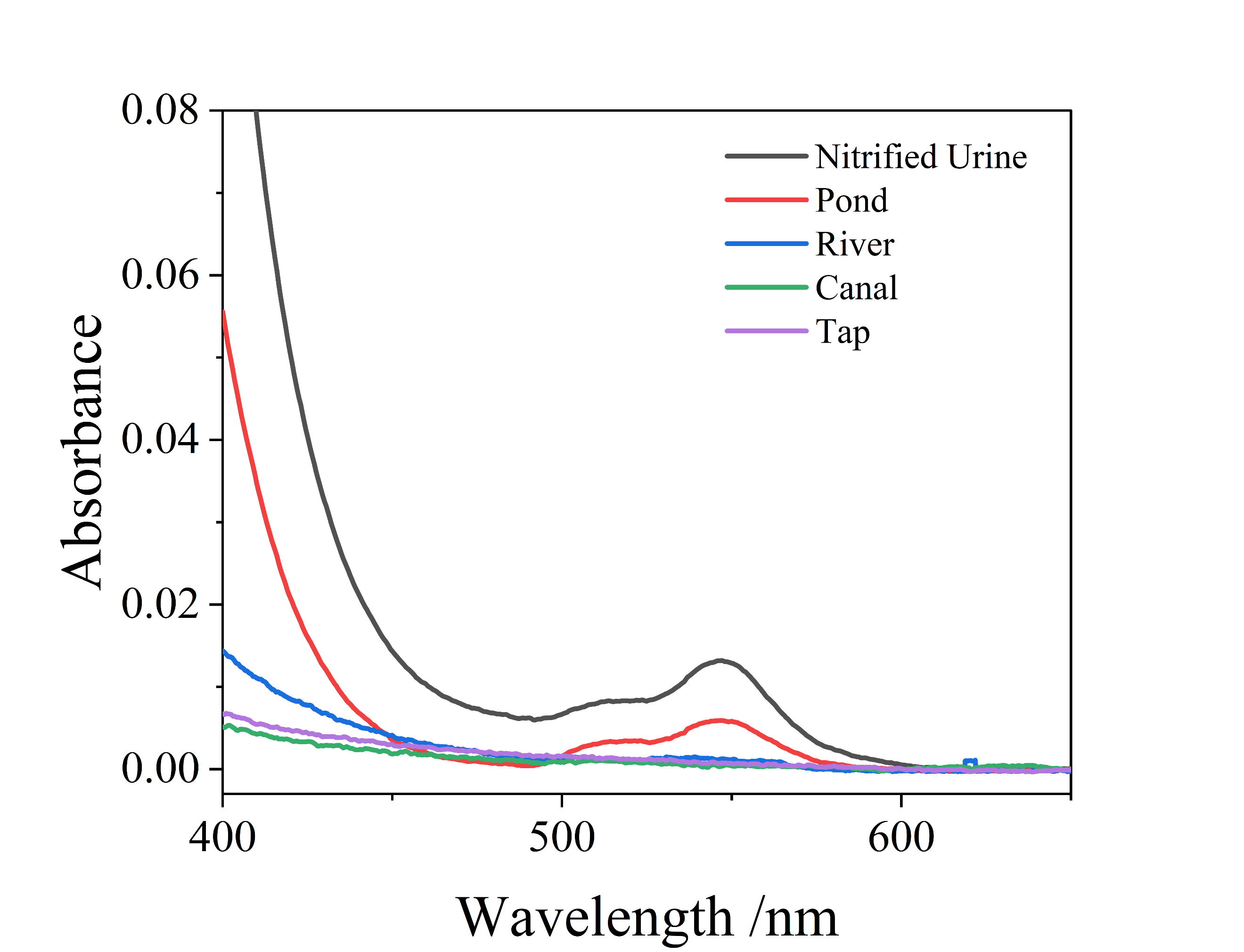

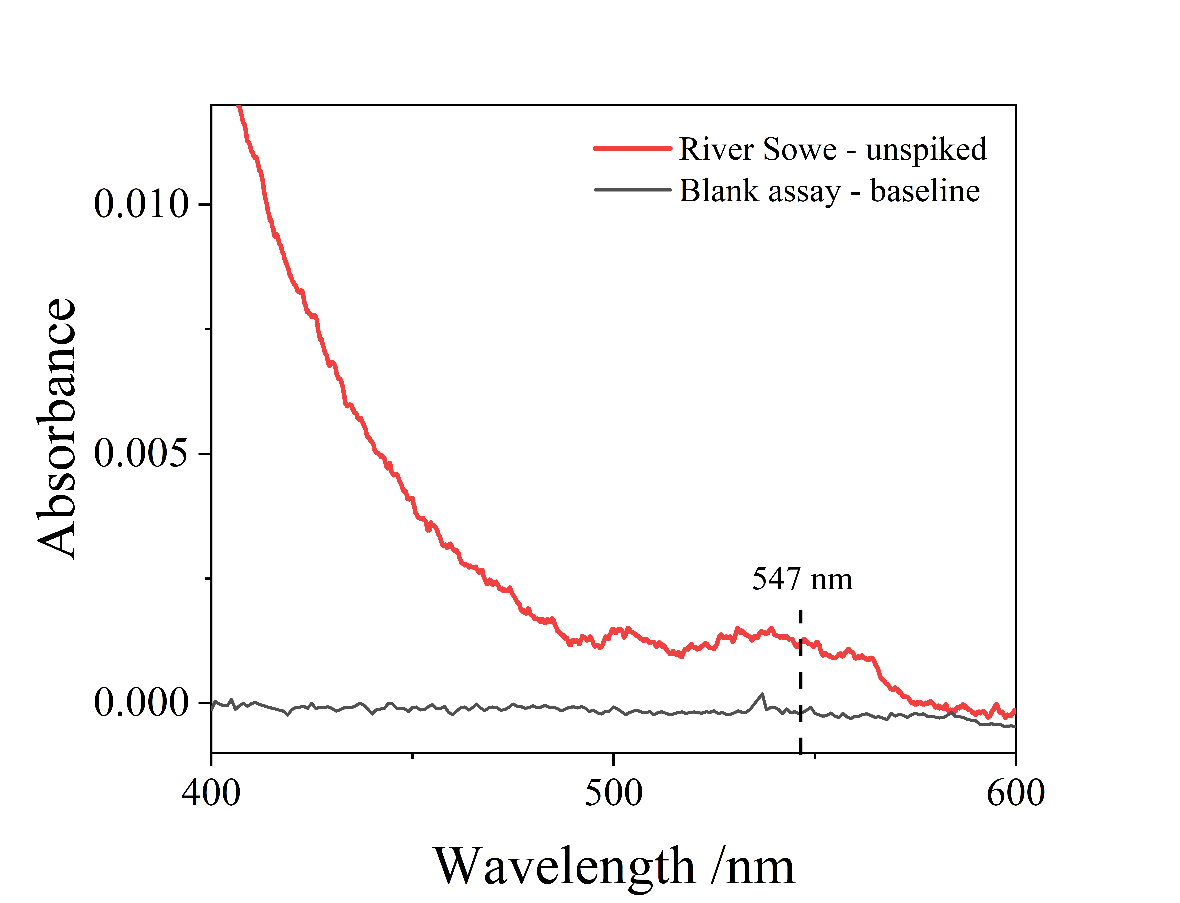

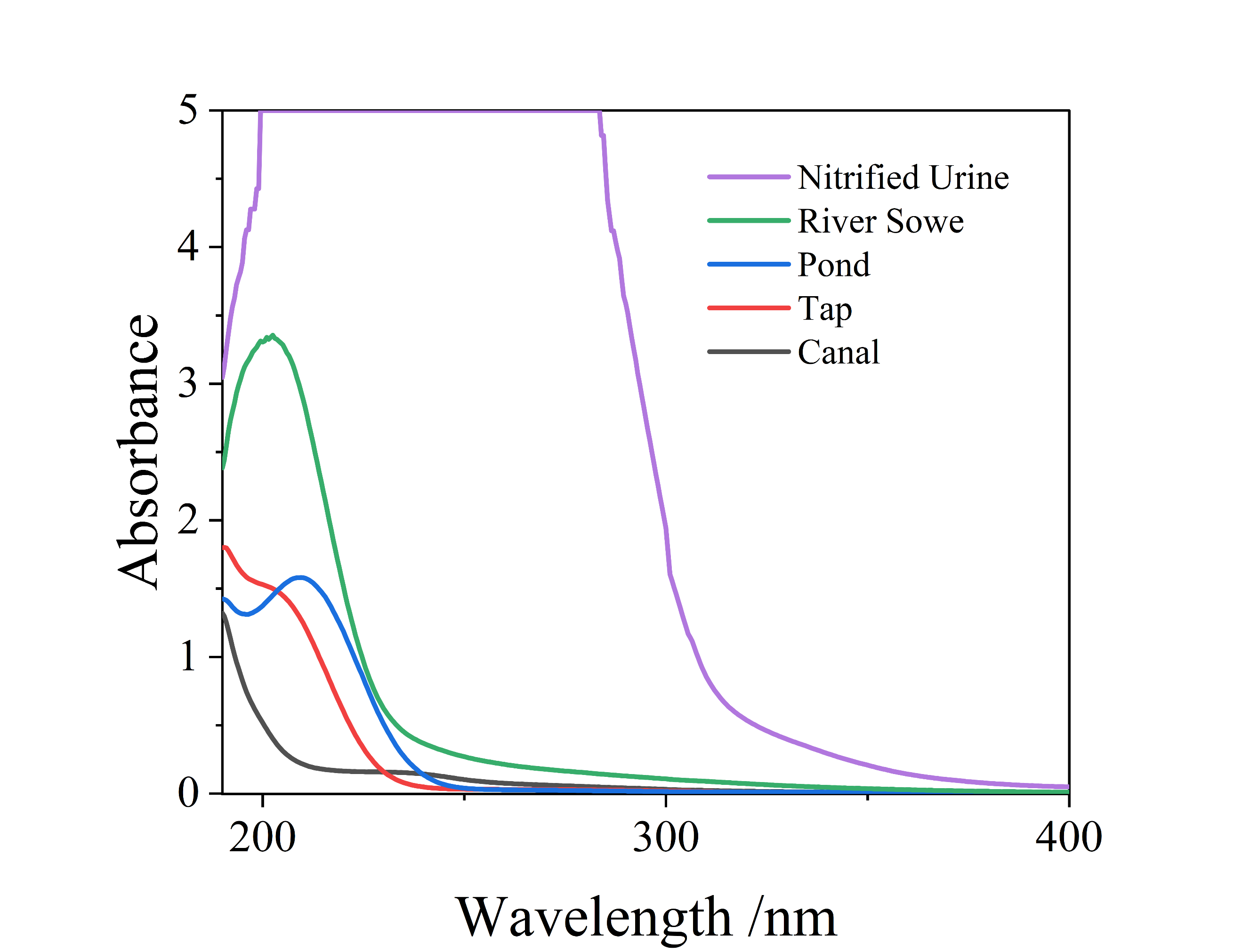


(a)

(b)

(c)

(d)

(e)


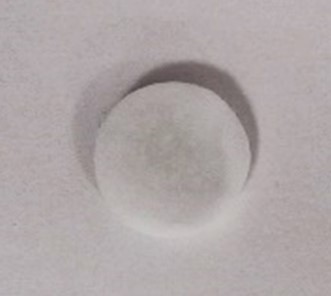


Figure S6. Top view (left) and side view (right) of the prepared nitrite assay pellet.


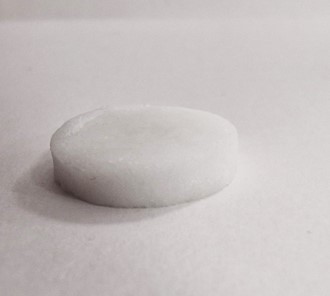

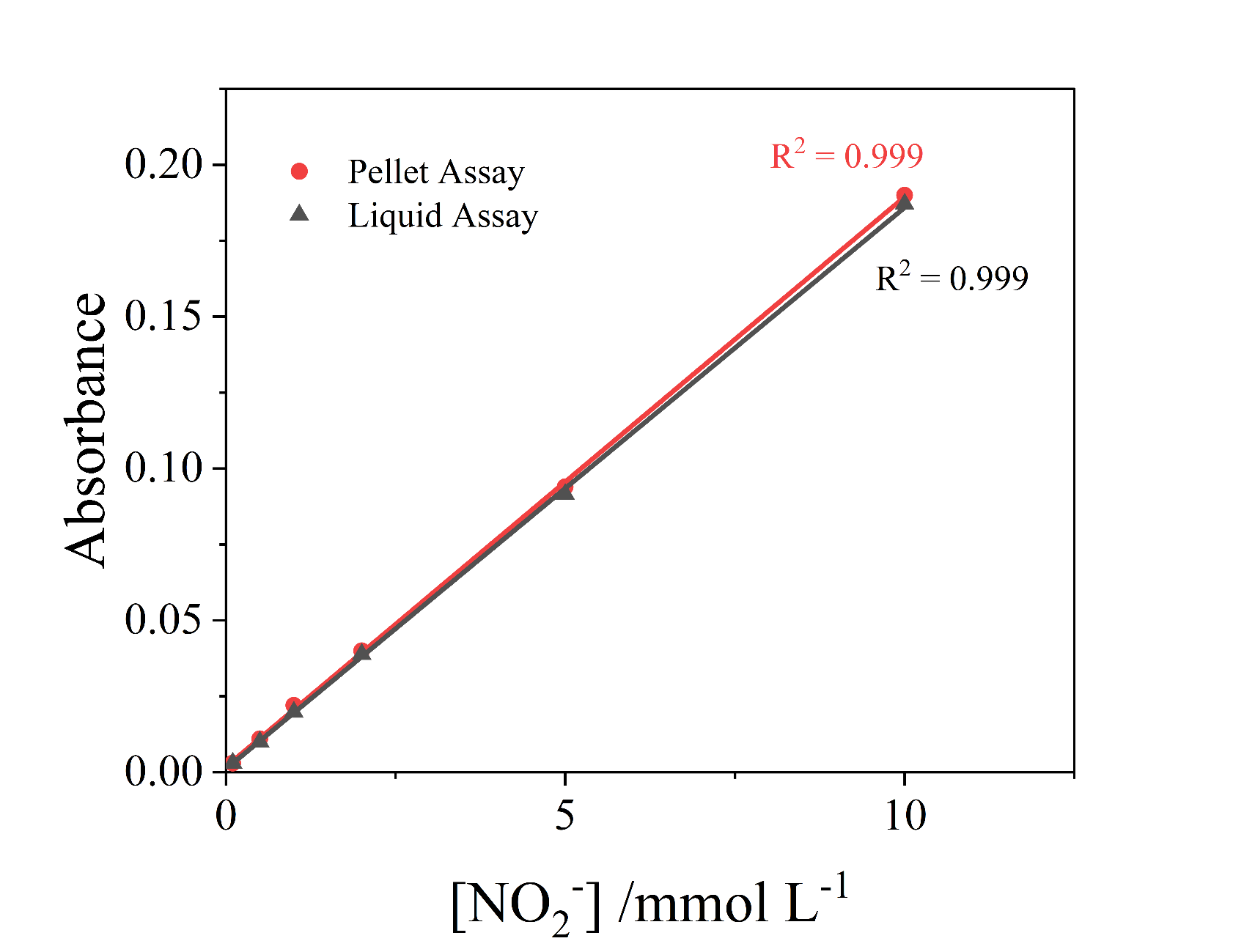


Figure S7. Standard calibration curve of the pellet (red symbol and line) and liquid (black symbol and line) assays, used for real sample analysis.

## Tables

Table S1. Figures of merit for the proposed nitrite detection assay.

| **Slope** | **Y-intercept** | **R^2^** | **Blank *σ_0_* (abs.)** | **Blank *σ_0_* (conc.)** | **LOD (mmol L^-1^)** | **LOQ (mmol L^-1^)** |
| --- | --- | --- | --- | --- | --- | --- |
| 0.01872 ± 1.3×10^-4^ | 0.00215 ± 6.2×10^-4^ | 0.99975 | 0.00018 | 0.00821 | 0.027 | 0.082 |

Table S2. Nitrite cuvette tests of real samples.

| **Analyte** | **Added NO_2_^-^ /mmol L^-1^** | **NO_2_^-^ Cuvette test /mmol L^-1^** |
| --- | --- | --- |
| **Tap** | 0 | < 0.03 |
|  | 1 | 1.01 ± 0.01 |
|  | 5 | 4.98 ± 0.06 |
| **Canal** | 0 | < 0.03 |
|  | 1 | 0.98 ± 0.02 |
|  | 5 | 5.11 ± 0.06 |
| **River** | 0 | < 0.03 |
|  | 1 | 1.02 ± 0.01 |
|  | 5 | 5.20 ± 0.07 |
| **Pond** | 0 | 0.09 ± 0.003 |
|  | 1 | 1.09 ± 0.02 |
|  | 5 | 5.13 ± 0.10 |
| **Nitrified urine** | 0 | 0.61 ± 0.06 |
|  | 1 | 1.63 ± 0.07 |
|  | 5 | 5.57 ± 0.12 |
